# Metaplasticity framework for cross-modal synaptic plasticity in adults

**DOI:** 10.3389/fnsyn.2022.1087042

**Published:** 2023-01-06

**Authors:** Hey-Kyoung Lee

**Affiliations:** The Solomon H. Snyder Department of Neuroscience, Zanvyl Krieger Mind/Brain Institute, Kavli Neuroscience Discovery Institute, Johns Hopkins University, Baltimore, MD, United States

**Keywords:** cross-modal plasticity, cortical plasticity, sensory experience, homeostatic synaptic plasticity, sliding threshold, LTP, LTD, adult plasticity

## Abstract

Sensory loss leads to widespread adaptation of neural circuits to mediate cross-modal plasticity, which allows the organism to better utilize the remaining senses to guide behavior. While cross-modal interactions are often thought to engage multisensory areas, cross-modal plasticity is often prominently observed at the level of the primary sensory cortices. One dramatic example is from functional imaging studies in humans where cross-modal recruitment of the deprived primary sensory cortex has been observed during the processing of the spared senses. In addition, loss of a sensory modality can lead to enhancement and refinement of the spared senses, some of which have been attributed to compensatory plasticity of the spared sensory cortices. Cross-modal plasticity is not restricted to early sensory loss but is also observed in adults, which suggests that it engages or enables plasticity mechanisms available in the adult cortical circuit. Because adult cross-modal plasticity is observed without gross anatomical connectivity changes, it is thought to occur mainly through functional plasticity of pre-existing circuits. The underlying cellular and molecular mechanisms involve activity-dependent homeostatic and Hebbian mechanisms. A particularly attractive mechanism is the sliding threshold metaplasticity model because it innately allows neurons to dynamically optimize their feature selectivity. In this mini review, I will summarize the cellular and molecular mechanisms that mediate cross-modal plasticity in the adult primary sensory cortices and evaluate the metaplasticity model as an effective framework to understand the underlying mechanisms.

## 1. Introduction

**FIGURE 1:**
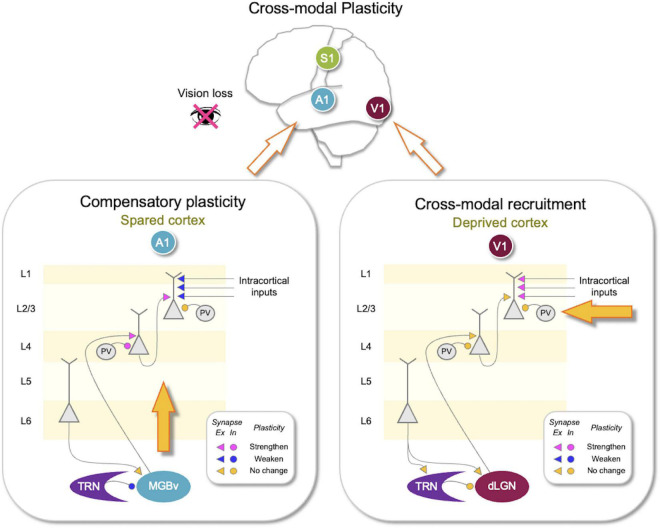
Synaptic changes associated with adult cross-modal plasticity. Sensory loss produces compensatory plasticity in the spared sensory cortices and cross-modal recruitment plasticity in the deprive sensory cortex. Lower panels show compilation of synaptic plasticity data from adult mice after vision loss (Petrus et al., 2014, 2015; Whitt et al., 2022). **(Lower right panel)** In the deprived primary visual cortex (V1), there is no plasticity of the feedforward (thalamocortical inputs to L4 and L4–L2/3) or the thalamic circuit, but lateral inputs to L2/3 potentiate. There is no change in parvalbumin (PV) inhibition onto L4 or L2/3 neurons. Corticothalamic synapses from V1 to thalamic reticular nucleus (TRN) or the primary visual thalamus [dorsal lateral geniculate nucleus (dLGN)] did not change. Adaptation of V1 circuit could allow cross-modal recruitment of V1 for processing other sensory inputs arriving through the potentiated lateral inputs to L2/3. **(Lower left panel)** In the spared primary auditory cortex (A1), there is potentiation of the feedforward synapses, including thalamocortical synapses and L4–L2/3 synapses. The strength of lateral inputs to L2/3 is weakened. Cortical inhibition mediated by PV neurons becomes stronger in L4 but does not change in L2/3. In addition, there is plasticity in the thalamic circuit, where TRN inhibition to the primary auditory thalamic nucleus [ventral portion of medial geniculate body (MGBv)] is reduced. Corticothalamic synapses to MGBv were not altered. Such adaptation of A1 is expected to amplify auditory signals and allow sharpening of tuning properties to mediate compensatory plasticity. Plasticity of synaptic strength following vision loss is color coded as shown in the insets. Pink, potentiated synapses; Blue, depressed synapses; Yellow: no change in synaptic strength. Triangles, excitatory (*Ex*) synapses; Circles, inhibitory (*In*) synapses.

Cross-modal plasticity refers to changes in brain function following a sensory loss that allows the spared senses to be used more effectively to guide behavior. There are two main changes: cross-modal recruitment of the brain areas that serve the lost sensory modality and compensatory plasticity of the brain areas that process the spared senses (Figure 1). Cross-modal recruitment has been observed in blind subjects where braille reading activates the deprived visual cortex (Sadato et al., 1996; Buchel et al., 1998; Burton et al., 2002b) and in deaf subjects where visual stimuli activate the deprived auditory cortex (Sandmann et al., 2012). This functional cross-modal recruitment of the deprived cortices is thought to enhance the processing of the remaining senses by increasing the cortical territory. This idea stems from the notion that cortical circuits are functionally equivalent and can process any sensory information presented to them. One of the supporting evidence for the functional equivalence of cortical circuits comes from a study, which rewired visual inputs to the primary auditory cortex (A1) of ferrets during early development causing visually guided behavior to become dependent on A1 (von Melchner et al., 2000). However, primary sensory cortices can be quite specialized in their anatomical organization, for example, barrel cortex in rodents and ocular dominance columns present in primary visual cortex (V1) of some carnivores and primates. Whether such anatomical specializations may affect the functional equivalence of cortical processing is unclear. In addition to the cross-modal recruitment, compensatory plasticity of the spared sensory areas is thought to allow refinement and increase the sensitivity of the spared sensory systems (Pascual-Leone and Torres, 1993; Sterr et al., 1998a,b; Roder et al., 1999; Elbert et al., 2002).

In animal models, early sensory loss results in cross-modal anatomical changes to the primary sensory cortices, especially of thalamocortical inputs (Henschke et al., 2018; Dooley and Krubitzer, 2019), but in adults, most of the cross-modal plasticity likely occurs through functional plasticity of pre-existing circuits (Lee and Whitt, 2015; Ewall et al., 2021). Cross-modal functional plasticity is observed in both the deprived and the spared primary sensory cortices (Figure 1). Evidence from animal studies suggests that these involve experience-dependent plasticity mechanisms based on Hebbian, both long-term potentiation (LTP) and long-term depression (LTD), and homeostatic mechanisms (Lee and Whitt, 2015; Ewall et al., 2021). In this mini-review, I will summarize the synaptic plasticity mechanisms of adult cross-modal plasticity and explain how utilizing the framework of the metaplasticity model can easily account for the global cortical adaptation in adults. To do this, I will first provide a brief introduction to the sliding threshold metaplasticity model.

## 2. Metaplasticity

**FIGURE 2:**
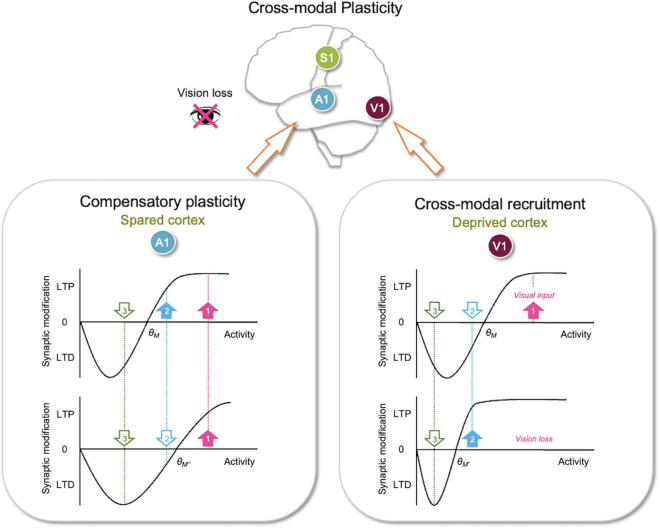
Metaplasticity sliding threshold model to explain adult cross-modal plasticity. Sliding threshold model can account for synaptic plasticity observed in the deprived sensory cortex (cross-modal recruitment) and the spared sensory cortices (compensatory plasticity). **(Lower right panel)** The sliding threshold model states that when past activity is low, the synaptic modification threshold (θm) slides down (from θ_*M*_–θ_*M′*_). This is expected to be the case in primary visual cortex (V1) with vision loss. Lower θm (θ_*M′*_) now allows the second strongest input (cyan, 2), which carries non-visual input, to potentiate. This may recruit V1 to process other sensory inputs in the absence of vision. The weakest input (green, 3) is still below the lowered θm (θ_*M′*_), hence stay in the LTD zone. This allows the neurons to maintain selectivity for the newly adopted feature (cyan, 2). **(Lower left panel)** According to the sliding threshold model, an increase in past activity slides up θm (from θ_*M*_–θ_*M″*_) to limit LTP induction and increase the range in which LTD can be induced. Vision loss in adults potentiates feedforward synapses in primary auditory cortex (A1) and reduces inhibition in the auditory thalamus (Figure 1), which is predicted to increase input activity to A1 L2/3 leading to a higher θm (θ_*M″*_). This causes activity from only the strongest input (pink, 1) to exceed the new θm (θ_*M″*_) to produce LTP, while the second strongest input (cyan, 2) now falls below the new θm (θ_*M″*_) and undergoes LTD. This in essence makes A1 neurons more selective, which would aid in the discrimination of auditory signals. Because the sliding threshold maintains a range for LTP and LTD, it is ideally suited to dynamically allow neurons to adopt new features in the absence of their main input to mediate cross-modal recruitment or become more feature selective when there is an increase in overall activity as would occur during compensatory plasticity. Filled upward pointing arrows, inputs with activity that surpasses θm, which will undergo LTP. Open downward pointing arrows, inputs with activity that falls below θm, which will undergo LTD.

Metaplasticity refers to the regulation of synaptic plasticity (Abraham and Bear, 1996) and often refers to the sliding threshold model proposed by Bienenstock, Cooper, and Monroe (BCM theory); a theoretical model that can provide stability to Hebbian plasticity (Bienenstock et al., 1982; Bear et al., 1987; Cooper and Bear, 2012). It was recognized that synaptic plasticity solely based on Hebbian mechanisms is limited and is unable to provide network stability that is necessary for neural function on its own. This is due to the fact that correlation based synaptic plasticity mechanisms, such as LTP and LTD, have in-built positive feedback. Strengthening synapse by LTP causes postsynaptic neurons to respond to inputs more strongly, which increases the coincidence of pre- and post-synaptic activity. This in turn promotes further LTP leading to over-excitability when left unchecked. Similar positive feedback occurs upon weakening synapses *via* LTD, but in the opposite direction that ultimately leads to inactivity. Such bistable property of Hebbian plasticity on neural network activity suggests a need for homeostatic control to provide stability. The sliding threshold model allows such homeostatic control by postulating that the induction threshold for LTP and LTD, referred to as the synaptic modification threshold (θm), slides as a function of the integrated past activity of a neuron (Figure 2). This key property of the sliding threshold allows neurons to dynamically tune their feature selectivity to the dominant input at the moment as a function of the history of overall activity (Bienenstock et al., 1982; Bear et al., 1987; Cooper and Bear, 2012). Here I will discuss the two main properties of the BCM model: the generation of feature selectivity and its dynamic regulation by past activity that endows homeostasis.

One of the main features of the sliding threshold model is the development of feature selectivity by LTP and LTD. Stronger inputs that exceed the θm undergo LTP, while weaker inputs that fail to produce sufficient postsynaptic activity are depressed *via* LTD. Thus neurons “select” the dominant input, at the expense of other weaker inputs. The ability of sensory cortical neurons to express feature selectivity is critical for sensory processing by enabling discrimination of distinct inputs to generate a percept of a certain feature in the sensory environment. While the initial setup of feature selectivity arises from the developmental organization of axonal projections based on guidance cues (Crowley and Katz, 1999, 2000), the refinement of feature selectivity is known to depend on the activity and in particular sensory experience (Sengpiel and Kind, 2002; Hooks and Chen, 2020). An essential feature of the sliding threshold is its ability to provide network stability, which is endowed by the regulation of θm as a function of the integrated past activity. If the neuron has been under high activity regime for a duration of time, θm increases (i.e., slides up) to make LTP more difficult to induce and promote LTD across the majority of synapses. Weakening the majority of the inputs *via* LTD causes the overall postsynaptic activity to decrease, hence providing homeostatic control of neural activity. In contrast, if the postsynaptic neuronal activity is low, θm is reduced to promote LTP and decreases the range of activity that produces LTD. While stabilizing the neural activity, the sliding threshold model preserves feature selectivity because stronger inputs will surpass θm to strengthen their connections at the expense of weaker inputs whether the θm is high or low. Implementing the sliding threshold to computational neural networks successfully explains the development of feature selectivity based on LTP/LTD while maintaining network stability (Bienenstock et al., 1982). As will be discussed later sliding threshold also enables dynamic regulation of the neuronal feature selectivity when there are changes to the type of inputs available to the neurons (Figure 2). The sliding threshold model was first experimentally demonstrated in V1, where reducing overall neural activity by dark rearing from birth promotes LTP and reduces LTD (Kirkwood et al., 1996). Since then, it has been confirmed across many brain regions (Lebel et al., 2001; Krucker et al., 2002; Solger et al., 2004; Muller et al., 2007; Narayanan and Johnston, 2010; Hulme et al., 2012) and malfunctioning of the sliding threshold has been reported in various neurological disease models (Hulme et al., 2013; Megill et al., 2015; Jang and Chung, 2016) suggesting its critical role in normal brain function.

At a molecular level, the sliding threshold is implemented by alterations in either the induction or the expression mechanisms of LTP/LTD. The initial postulate of the sliding threshold model stated a horizontal shift of the synaptic modification function (Figure 2), but now there is evidence that the synaptic modification function could shift vertically (Seol et al., 2007; Huang et al., 2012; Hong et al., 2020). Both the horizontal and vertical shift in the synaptic modification function slides the θm in the same manner, but the molecular mechanisms underlying the two are distinct. The horizontal shift in synaptic modification occurs by changes in the induction mechanisms of LTP/D, which in molecular terms has been demonstrated as changes in N-methyl-D-aspartate (NMDA) receptor (NMDAR) subunit composition. Low neuronal activity upregulates the expression of NMDAR GluN2B (or NR2B) subunit (Quinlan et al., 1999; Philpot et al., 2001, 2003), which confers longer current duration compared to GluN2A (or NR2A) containing NMDARs (Chen et al., 1999; Rumbaugh and Vicini, 1999). This in essence allows larger intracellular Ca^2+^ increase through NMDARs to promote LTP. With heightened neuronal activity, GluN2A becomes the dominant subunit for NMDARs, which increases the θm to make LTP induction more stringent. In contrast, the vertical shift in synaptic modification (also referred to as the Pull-Push model) has been shown to occur with neuromodulators acting on LTP/LTD expression mechanisms (Seol et al., 2007; Huang et al., 2012; Hong et al., 2020; Mihalas et al., 2021). Neuromodulators linked to cyclic adenosine 3′,5′-monophosphate (cAMP) signaling promote LTP by increasing phosphorylation of α-amino-3-hydroxy-5-methyl-4-isoxazolepropionic acid receptors (AMPARs), especially on the protein kinase A target serine-845 (S845) residue of the GluA1 subunit to promote LTP (Seol et al., 2007; Qian et al., 2012). GluA1-S845 phosphorylation has been shown to increase the content of cell surface AMPARs (Oh et al., 2006; He et al., 2011) and prime AMPARs for synaptic recruitment by LTP (Lee and Kirkwood, 2011; Diering and Huganir, 2018). In contrast, neuromodulators linked to phospholipase C (PLC) slide up the θm by promoting LTD and preventing LTP expression (Huang et al., 2012). The main difference between the horizontal and vertical shift in synaptic modification function is that the latter puts synapses into an LTP-only or an LTD-only mode while the former preserves the full range of LTP and LTD (Figure 2).

Next, I will review the synaptic mechanisms of cross-modal plasticity in the adult primary sensory cortices and discuss how the horizontal shift in synaptic modification function could account for the observed findings.

## 3. Cross-modal synaptic plasticity in adult primary sensory cortices

In the adult primary sensory cortices, the superficial layers (L2/3) are likely a major substrate for adult cross-modal plasticity. L2/3 synapses retain their ability to undergo LTP and LTD as well as homeostatic synaptic plasticity into adulthood (Goel and Lee, 2007; Jiang et al., 2007). This contrasts plasticity in L4, where thalamocortical LTP/LTD and homeostatic synaptic plasticity display a short early critical period for plasticity (Crair and Malenka, 1995; Feldman et al., 1998; Desai et al., 2002; Jiang et al., 2007; Barkat et al., 2011; Rodriguez et al., 2018). Moreover, the functional connectivity of the L2/3 neurons is well poised to integrate top-down contextual information with the bottom-up sensory responses from thalamorecipient neurons in L4. Besides feedforward inputs from L4, L2/3 principal neurons receive long-range inputs from multisensory association cortices and higher order sensory cortices, direct connections from other primary sensory cortices, as well as inputs from higher order thalamic nuclei that carry multisensory information (Ewall et al., 2021).

### 3.1. Synaptic plasticity of the deprived sensory cortex

Even a short duration of sensory loss impacts normal development (Sengpiel and Kind, 2002; Hooks and Chen, 2020), but there is some degree of plasticity in adult sensory cortices (Lee and Whitt, 2015; Ribic, 2020), especially in the superficial layers. For example, a couple of days of complete visual deprivation leads to homeostatic strengthening of excitatory synapses measured as miniature excitatory postsynaptic currents (mEPSCs) in adult V1 L2/3 neurons (Goel and Lee, 2007). These changes can be interpreted in the framework of synaptic scaling (Turrigiano and Nelson, 2004; Turrigiano, 2008) or sliding threshold (Ewall et al., 2021). Synaptic scaling model would allow network activity (i.e., firing rate) homeostasis that can prevent overall silencing of the cortical network caused by lost inputs, while sliding threshold would enable dynamic remapping of neuronal features in addition to activity homeostasis. Synaptic scaling and sliding threshold likely operate across different activity regimes to maintain network homeostasis (Lee and Kirkwood, 2019).

Key distinguishing features of synaptic scaling and sliding threshold model are dependence on activity, especially of NMDARs, and input-specific nature of plasticity. Inactivity-driven synaptic scaling was first demonstrated in neuronal cultures upon pharmacological blockade of activity using tetrodotoxin (TTX) or AMPAR blockers (O’Brien et al., 1998; Turrigiano et al., 1998). Subsequent studies demonstrated that NMDAR blockade can accelerate the rate of scaling up synapses with TTX (Sutton et al., 2006). In contrast, according to the sliding threshold model, while the sliding of the θm can happen in the absence of activity, the manifestation of changes in AMPAR-mediated synaptic transmission requires LTP or LTD that is dependent on NMDAR activity (Cooper and Bear, 2012). It is demonstrated that the potentiation of mEPSCs in V1 L2/3 following visual deprivation is blocked by NMDAR antagonist application (Rodriguez et al., 2019) and in particular by blockers of GluN2B (Bridi et al., 2018). These data support the sliding threshold model and are at odds with the synaptic scaling model. Furthermore, the potentiation of mEPSCs in V1 L2/3 neurons following visual deprivation is not multiplicative across all synapses in adults (Goel and Lee, 2007) and is specific to lateral inputs but not observed at feedforward inputs from L4 (Petrus et al., 2015; Chokshi et al., 2019; Figure 1). These results corroborate that in intact cortical circuits with distinct inputs that carry different patterns of activity, homeostatic adaptation is not uniform across all the synapses. Such input-specific and NMDAR-dependent plasticity supports the sliding threshold model, but we cannot exclude the role of synaptic scaling especially with extreme changes in activity (Lee and Kirkwood, 2019).

According to the sliding threshold model, loss of a major input, such as visual input to V1 neurons, is expected to decrease the overall neuronal activity (Figure 2). If this persists, the theory states that θm will slide down, which allows a subset of previously weak inputs to cross the lowered θm and strengthen *via* LTP. This allows V1 neurons, devoid of visual inputs, to adopt these newly potentiated inputs as their main driver. Inputs with activity below the new θm will still undergo LTD, which permits V1 neurons to maintain selectivity to the newly adopted inputs. Therefore, the sliding threshold model enables neurons to dynamically adopt new features based on the changes in the landscape of sensory experience. Particularly for cross-modal plasticity, the metaplasticity model allows V1 neurons to respond to non-visual activity carried by the newly potentiated inputs. This could serve as a substrate for cross-modal recruitment observed in blind subjects (Sadato et al., 1996; Buchel et al., 1998; Burton et al., 2002a; Sandmann et al., 2012). While the main function of the primary sensory cortices is to process their respective sensory information, multisensory information is readily available at this early stage of sensory processing. Indeed, *in vivo* whole-cell recordings demonstrate subthreshold functional connections between primary sensory cortices (Iurilli et al., 2012). Furthermore, these subthreshold cortico-cortical connections provide multisensory modulation of the primary inputs. For example, loud sound sharpens the orientation tuning of L2/3 V1 neurons *via* direct input from A1 L5 neurons (Ibrahim et al., 2016; Deneux et al., 2019). Conceivable then, visual deprivation-induced sliding down of θm in V1 L2/3 neurons could allow these subthreshold A1 inputs to potentiate, thus enabling recruitment of V1 for auditory processing.

### 3.2. Synaptic plasticity of the spared sensory cortices

Plasticity of the spared primary sensory cortices is thought to refine the processing of the spared sensory inputs, improving discrimination, and detection. At a cellular level, cross-modal sensory loss strengthens feedforward connections of spared primary sensory cortices (Figure 1). For example, depriving vision of adult mice potentiates the thalamocortical synapses in L4 and L4–L2/3 synapses in A1 (Petrus et al., 2014, 2015) and potentiates L4–L2/3 synapses in the rat barrel cortex (Jitsuki et al., 2011). Similarly, deafening adult mice strengthens thalamocortical synapses in V1 L4 (Petrus et al., 2014; Rodriguez et al., 2018). The potentiation of thalamocortical synapses in adults is of interest because both thalamocortical LTP and LTD were shown to be restricted to an early postnatal critical period (Crair and Malenka, 1995; Feldman et al., 1998; Desai et al., 2002; Jiang et al., 2007; Barkat et al., 2011). Cross-modal potentiation of thalamocortical synapses is likely to occur *via* recovery of NMDAR-dependent thalamocortical LTP in adults (Rodriguez et al., 2018), but the mechanism is currently unknown.

L4–L2/3 synapses, unlike thalamocortical synapses, retain plasticity in adults (Goel and Lee, 2007; Jiang et al., 2007). The potentiation of the feedforward inputs in the spared sensory cortices suggests that feedforward activity increases to a level above θm to produce LTP. For cross-modal plasticity in A1, the evidence suggests a central mechanism that may amplify auditory activity. This may be achieved by a targeted reduction in thalamic inhibition (Whitt et al., 2022). Specifically, depriving vision of adult mice caused a selective reduction of thalamic reticular nucleus (TRN) inhibition to the auditory thalamus [ventral portion of medial geniculate body (MGBv)] but not to the visual thalamus [dorsal lateral geniculate nucleus (dLGN)] (Figure 1). Such reduction in inhibition is expected to increase feedforward activity to A1, which would exceed the θm to induce LTP of feedforward synapses (Figure 2).

Concomitantly, cross-modal sensory deprivation reduces the strength of lateral intracortical synapses to L2/3 neurons (Petrus et al., 2015; Figure 1). The cross-modal synaptic depression of the lateral inputs in L2/3 of the spared sensory cortices can be explained by an increase in θm that results from enhanced feedforward activity (Figure 2). Cross-modal sensory deprivation-induced increase in θm would induce LTD in the majority of inputs as their activity will now fall within the LTD range below the new θm. This theoretically can explain the observed depression of the lateral excitatory inputs (Petrus et al., 2015), which by definition will produce weaker activity compared to the feedforward inputs. At a functional level, the decrease in the θm results in enhanced feature selectivity of the neurons in the spared sensory cortices, because only the few highly active inputs will cross the θm to remain strengthened to drive postsynaptic firing (Figure 2). The spared cortical circuit also exhibits increased inhibition from parvalbumin (PV) interneurons (Petrus et al., 2015), which will aid in the sharpening of the feature selectivity.

## 4. Conclusions

The sliding threshold model is compatible with experimental observations of synaptic plasticity related to both cross-modal recruitment and compensatory plasticity. Viewing cross-modal plasticity in the framework of sliding threshold presents testable predictions. Because the synaptic plasticity is ultimately driven by NMDAR-dependent Hebbian modification in accordance with the newly defined synaptic modification threshold, it suggests that cross-modal plasticity will be dependent on sensory experience in the spared modalities. Such requirement of experience may explain the varied observations of the outcome of cross-modal plasticity in human subjects, especially when a sensory modality is lost later in life (Frasnelli et al., 2011; Lazzouni and Lepore, 2014). Furthermore, observations that pre-existing functional connections across the sensory cortices remain plasticity even in adults suggest that cross-modal plasticity mechanisms could be used for enhancing adult brain function.

## 5. Nomenclature

A1, primary auditory cortex; AMPAR, α-amino-3-hydroxy-5-methyl-4-isoxazolepropionic acid receptor; cAMP, cyclic adenosine 3′,5′-monophosphate; dLGN, dorsal lateral geniculate nucleus; GluN2A, glutamate ionotropic receptor NMDA type 2A; GluN2B, glutamate ionotropic receptor NMDA type subunit 2B; L2/3, layer 2/3; L4, layer 4; L5, layer 5; LTD, long-term depression; LTP, long-term potentiation; mEPSCs, miniature excitatory postsynaptic currents; MGBv, ventral portion of medial geniculate body; NMDAR, N-methyl-D-aspartate (NMDA) receptor; NR2A, NMDAR 2A subunit; NR2B, NMDAR 2B subunit; PLC, phospholipase C; TRN, thalamic reticular nucleus; TTX, tetrodotoxin; V1, primary visual cortex; θm, synaptic modification threshold.

## Author contributions

H-KL synthesized and conceptualized the ideas and wrote the manuscript.
